# Cancer Stigma Among 800 Saudi Citizens: A Cross-Sectional Study and Literature Review

**DOI:** 10.7759/cureus.49088

**Published:** 2023-11-20

**Authors:** Hanan A Albenayyan, Renad AlSubaie, Maryam O Alarfaj, Lames Alshekhmobarak, Mohammed F Alkhalifah, Hajar Alsaleem, Dalal Almulhim, Aisha A AlJughaiman, Fatimah A Albahrani, Almaha A Aleidan, Razan M Alzahrani, Lama Alobaid, Taghreed Alhinidi

**Affiliations:** 1 Medicine, King Faisal University, Al Ahsa, SAU; 2 Radiation Oncology, King Fahad Specialist Hospital, Dammam, SAU

**Keywords:** oncology, general population, cass, stigma, cancer

## Abstract

Introduction: Cancer-related stigmatization is a noteworthy phenomenon, yet it has not received sufficient attention in public health studies. Despite recent advancements in treatment and improvements in survival, the burden of stigma remains a challenging concern for individuals diagnosed with cancer.

Aim: This study aims to assess the presence of cancer stigma in the Saudi Arabian population by using the Cancer Stigma Scale (CASS).

Methods: A cross-sectional study was conducted among the general population of Saudi Arabia. Data collection was facilitated through a self-administered online questionnaire, incorporating socio-demographic variables such as age, gender, and regional residence and employing the CASS instrument to gauge the prevalent attitudes and stigmas related to cancer.

Results: Out of the 874 participants, a majority of 87.1% were female, with 60.2% aged between 20 and 39 years. Notably, 59% reported having a close friend or family member diagnosed with cancer. The average CASS score stood at 1.59 (SD 0.39) on a 5-point scale, with an overwhelming 97.1% registering scores under 2.5, suggesting a generally low stigma perception. In dissecting the CASS components, 'severity' recorded the highest mean score (mean: 2.23), followed by 'awkwardness' (mean: 1.86) and 'financial discrimination' (mean: 1.71). 'avoidance' registered the lowest mean score at 1.11. Notably, a trend of increasing stigma was observed with advancing age, and male respondents indicated a marginally higher propensity towards stigmatizing attitudes.

Conclusion: In Saudi Arabia, cancer-related stigma is generally low. However, 'severity' is the most prominent stigma aspect, with 'avoidance' being the least. Older individuals and males exhibit slightly higher stigmatizing attitudes. These insights highlight the need for targeted public health efforts to address remaining stigmatization, especially based on age and gender.

## Introduction

Cancer is the rapid growth of abnormal cells beyond their normal limits, which can invade nearby body parts and spread to other organs [1]. There are more than a hundred different types of cancer. In 2020, there were an estimated 18.1 million cancer cases worldwide. Of these, 9.3 million cases affected men, and 8.8 million cases affected women. The incidence of all cancers in Saudi Arabia, except melanoma, has been estimated at 17,522 cases (8,296 males and 9,226 females) [2]. The most common cancers in Saudi Arabia are breast, colorectal, prostate, brain, lymphoma, kidney, and thyroid. Their prevalence rates are as follows: breast cancer at 53%, colon cancer at 50.9%, prostate cancer at 42.6%, and thyroid cancer at 12.9%. Saudi Arabia ranks second in cancer mortality rates among all Arabian Gulf countries. The estimated mortality was 9,134 cases across all ages and genders [3].

One of the most common causes of death worldwide is cancer. However, many of these fatalities are preventable. Healthy lifestyle decisions such as smoking cessation and getting immunization against cancer-causing pathogens can prevent between 30% and 50% of cancers [4]. People have a generally negative attitude towards cancer, with most people viewing it as a terrifying illness leading to death [5]. The utterance of the word "cancer" evokes considerable apprehension in numerous individuals, as they hold the belief that cancer is an ailment devoid of remedy and heralds an imminent fatality [6]. When someone is diagnosed with cancer, fear and a fatalistic view of the disease as a "killer" is acknowledged [7,8]. The unwillingness of individuals who have battled or survived cancer to talk about it in their community has been noted as another problem [9]. As a result, the community primarily pays attention to the negative aspects of cancer, while contrary to the belief, cancer is a treatable and curable disease in many cases with appropriate intervention.

Such social stigma can instill concern and preclude people from using services for early cancer detection or screening or, at the very least, receiving cancer treatments. Stigma is a social phenomenon that can be both experienced and anticipated by a person [10]. This happens when aspects of labeling, stereotyping, separation, status loss, and discrimination take place in a power position that permits them to occur [11]. Stigmatization of a disease is referred to as health-related stigma and can be ascribed to a single person with the disease, a group of people who share the disease, or the disease more broadly [12]. It is "marked by exclusion, rejection, blame, or devaluation that arises from experience, perception, or reasonable prediction of a negative social judgment about a person or group identified with a particular health problem [13]. For those who are stigmatized, stigma and its psychosocial effects result in unbearable misery [14]. Additionally, stigma has indirect but detrimental effects on public health initiatives to combat the diseases in question [15].

Cancer is a well-known factor of stigmatization in society but has not been properly addressed in public health studies. Despite recent advancements in treatment and improvements in survival, feelings of stigma are a problematic concern among cancer patients. Due to the presence of early cancer detection methods (screening), people must take the possibility of a cancer diagnosis into account when making preventive health decisions [6]. Fear of stigmatization has been identified as a potential impediment to self-examination, screening, and the delayed presentation of cancer symptoms [16,17]. Because some people might be deterred from participating in cancer prevention or early detection due to expectations and a fear of being stigmatized [18,19]. Public stigma of cancer may have a detrimental effect on attempts to improve public health and lessen the burden of cancer on society as a whole, in addition to having an adverse effect on cancer patients [20]. This includes delayed diagnosis and treatment, which increases the danger of disease transmission in cases of infectious diseases and worsens the prognosis for recovery in the majority of disorders [21]. This paper represents the first study that shows the cancer stigma in Saudi Arabia, assessed by the Cancer Stigma Scale (CASS).

## Materials and methods

Study design and sampling technique

This study is a descriptive, cross-sectional study conducted with the objective of understanding the stigmatization related to cancer in the Kingdom of Saudi Arabia. A random sampling method was used, where an electronic questionnaire was disseminated to a diverse audience via various social media platforms to ensure wide reach and representation. The target population consisted of residents of Saudi Arabia aged 12 years and above. Given the country's diverse population and wide age range, this ensured a comprehensive overview of the cancer stigma across different age groups and regions.

To determine the sample size, the following formula was utilized. With a confidence interval set at 95% and a margin of error at 5%, a sample size of 382 individuals was deemed representative of the study's population.

= P(1-P) (Z/)2 (E)2

Inclusion and exclusion criteria

Residents of all regions of Saudi Arabia, aged from 12 years old to more than 60 years old, were included in the selection criteria. All participants younger than 12 years, participants not from Saudi Arabia, and participants who did not fully answer the questionnaire were all excluded.

Statistical analysis

Data were processed and analyzed using the Statistical Package for Social Sciences (SPSS) software, version 26.0 (IBM Corp., Armonk, NY). Categorical variables were represented as numbers and percentages, whereas continuous variables were summarized using mean and standard deviation.

Before any comparisons were made, the normality of data distribution was verified using both the Shapiro-Wilk and Kolmogorov-Smirnov tests. Given the non-normal data distribution, non-parametric tests were utilized. The Mann-Whitney Z-test and Kruskal Wallis H-test were employed to identify significant differences in the CASS scores concerning the socio-demographic variables. Furthermore, the Pearson correlation coefficient was applied to gauge the correlation between the overall CASS score and its individual domains. A p-value below 0.05 was taken as the threshold for statistical significance.

Measurement tools

The Cancer Stigma Scale (CASS) was the primary tool utilized. Originating from England in 2010, this validated questionnaire delves into six distinct aspects of the stigma associated with cancer: Awkwardness, Avoidance, Perceived Severity, Policy Opposition, Personal Responsibility, and Financial Discrimination. Specific sections like awkwardness assess societal comfort levels around cancer patients, while perceived severity evaluates perceptions around the impact and prognosis of cancer. Notably, elements like policy opposition tie closely with financial discrimination, indicating societal views on resource allocation for cancer treatment and patient support.

Ethical considerations

Prior to the commencement of the study, ethical approval was obtained from the Institutional Research Board and the Ethics Committee of King Faisal University in Al-Ahsa (Approval Code: KFU-REC-2022-DEC-ETHICS408). Ensuring the privacy of the participants was paramount; thus, the questionnaire was designed to exclude any personally identifiable information. All collected data were stored in SPSS format with no links to individual respondents, ensuring complete anonymity.

## Results

This study enrolled 874 participants. The most common age group was 20 to 39 years old (60.2%) with nearly all being female (87.1%). Respondents who lived in the Central Region constitute 33.3%. Most of the respondents held bachelor’s degrees or higher (62.2%). A family or friend history of cancer was reported by 59% (Table 1).

**Table 1:** Socio-demographic characteristics of participants (n=874)

Study Data	N (%)
Age group	
12 – 19 years	258 (29.5%)
20 – 39 years	526 (60.2%)
40 – 59 years	75 (08.6%)
≥60 years	15 (01.7%)
Gender	
Male	113 (12.9%)
Female	761 (87.1%)
Residence region	
Eastern Region	248 (28.4%)
Central Region	291 (33.3%)
Western Region	187 (21.4%)
Northern Region	67 (07.7%)
Southern Region	81 (09.3%)
Educational level	
Secondary school	12 (01.4%)
High school	257 (29.4%)
Diploma	61 (07.0%)
Bachelor or above	544 (62.2%)
Family/Friend history of cancer	
Yes	516 (59.0%)
No	358 (41.0%)

Regarding the descriptive statistics of CASS domains, the mean scores of severity, awkwardness, financial discrimination, policy opposition, personal responsibility, and avoidance were 2.23, 1.86, 1.71, 1.39, 1.26, and 1.11, respectively. The overall mean score of CASS was 1.59 (SD 0.39). In addition, we found that there was a significant positive correlation between the total CASS score among its domains including severity (r=0.617), awkwardness (r=0.639), financial discrimination (r=0.564), policy opposition (r=0.484), personal responsibility (r=0.568) and avoidance (r=0.529). This further indicates that the increase in the score of each stigma domain is correlated with the increase in the overall cancer stigma (Table 2).

**Table 2:** Descriptive statistics and correlation of the Cancer Stigma Scale (CASS) (n=874) ** Correlation is significant at p<0.05 level.

CASS domains	Score Mean ± SD	CASS score r-value
Severity	2.23 ± 0.96	0.617 **
Awkwardness	1.86 ± 0.82	0.639 **
Financial discrimination	1.71 ± 0.83	0.564 **
Policy opposition	1.39 ± 0.58	0.484 **
Personal responsibility	1.26 ± 0.55	0.568 **
Avoidance	1.11 ± 0.37	0.529 **
Total CASS	1.59 ± 0.39	1

When measuring the differences in the score of CASS according to the socio-demographic characteristics of participants (Table 3), it was found that increasing age was associated with increasing CASS score (H=8.075; p=0.018) while male gender was more associated with having a higher CASS score (Z=1.985; p=0.047).

**Table 3:** Differences in CASS score in relation to socio-demographic characteristics of participants (n=874) CASS: Cancer Stigma Scale ^a^P-value has been calculated using the Kruskal Wallis H-test; ^b^P-value has been calculated using Mann Whitney Z-test; ** Significant at p<0.05 level.

Factor	CASS Score Total (5) Mean ± SD)	Z/H-Test	P-value
Age group ^a^			
12 – 19 years	1.54 ± 0.36	H=8.075	0.018 **
20 – 39 years	1.61 ± 0.40		
≥40 years	1.69 ± 0.43		
Gender ^b^			
Male	1.66 ± 0.41	Z=1.985	0.047 **
Female	1.59 ± 0.39		
Residence region ^a^			
Eastern Region	1.62 ± 0.39	H=2.809	0.422
Central Region	1.58 ± 0.37		
Western Region	1.59 ± 0.37		
Northern/Southern Region	1.59 ± 0.47		
Educational level ^b^			
Diploma or below	1.59 ± 0.40	Z=0.318	0.751
Bachelor or above	1.59 ± 0.39		
Family/Friend history of cancer ^b^			
Yes	1.59 ± 0.39	Z=0.399	0.690
No	1.60 ± 0.39		

## Discussion

Cancer stigma is usually measured with the Cancer Stigma Scale (CASS). In different studies carried out in different countries, it was shown that cancer stigma is generally high; however, many factors could contribute to the variation between the countries. For example, it showed that the stigmatization of cancer among English adults is generally low. However, among men and those of non-English ethnicity, cancer stigma was higher [22]. Similarly, another study that was conducted in Malaysia among university students showed a low level of stigmatization toward cancer, and men in that study also had a higher stigma level toward cancer. One study on stigma towards mental illness explained that women have lower stigma because of their sensitivity to the horrifying effects of stigma due to their high level of empathy and prejudice [23]. Also, as women are involved more in cancer campaigns and screening, they are more likely to have lower stigma [24].

Generally, it’s expected that socio-demographic factors play a role in stigma components, and some differences could be influenced by differences in cultures, religions, and lifestyles [25,26]. Moreover, in England, the majority disagreed with the statements of avoidance, awkwardness, and personal responsibility, but supported the statements regarding the acceptability of financial discrimination, policy opposition, and severity of a cancer diagnosis [21]. Similarly, in Malaysia, it was found that statements of awkwardness, avoidance, and financial discrimination have the lowest endorsement. On the other hand, statements about cancer severity, policy opposition, and personal responsibility have higher endorsement [23]. In both studies, the subscale with the lowest mean score was avoidance [22,24]. It has been shown that being in contact with cancer patients could differ the response on the subscales [25]. One study that was done in India supported the previous statement, as cancer-related stigma was common among patients and caregivers [27]. Comparably, in another study in Korea that was conducted among cancer patients, nearly one-third of the participants had stigmatization toward cancer, and unfortunately, among these patients, depression was 2.5 times more likely [28]. Moreover, the level of education also plays an important role in cancer stigmatization, as people with a higher level of education were found to have a lower level of stigma [23,28]. Previous points explain the overall low level of cancer stigma, as education level is becoming increasingly important worldwide and educated people globally [29].

The findings of our study revealed that using the CASS questionnaire, cancer stigma among the Saudi general population was low. The overall mean score of CASS was 1.59 (SD 0.39) and approximately 97.1% had a score of fewer than 2.5 points suggesting a minimal cancer stigma. This finding is consistent with that of Vrinten et al. [20]. According to their reports, there was a low cancer stigma among the English population. However, from the perspective of caregivers and patients [28], a higher level of cancer stigma was documented. Findings suggest that approximately 60% of patients were suffering from cancer stigma and more than one-third of both patients and their caregivers were experiencing internalized stigma, and it showed a moderate association with feeling nervous and anxious, uncontrolled worrying, and feelings of depression and hopelessness, while in Iran [29], 26.1% had negative attitudes towards cancer stigma scoring 2.5 or higher. In our study, only 2.9% of the subjects were categorized as having a high level of stigma; however, it has to take into consideration the studied population since cancer patients may have been suffering greater stigma than the general population due to the significant burden given by the underlying disease.

Regarding cancer stigma domains, out of 5 total score points, cancer severity came out with the highest mean score (mean score: 2.23) and using a cutoff point of ≥2.5, 37.3% were considered to have a high level of agreement to severity domain, followed by awkwardness (mean score: 1.86; 22% high level), financial discrimination (mean score; 1.71; 14.5% high level), policy opposition (mean score: 1.39; 4.5% high level), and personal responsibility (mean score: 1.26; 3.1% high level) while avoidance came out the least level of agreement (mean score: 1.11; 2.1% high level). Consistent with our reports, Vrinten and his colleagues [20], detected the severity of the cancer diagnosis as the item with the highest level of agreement, followed by the items of acceptability of making financial decisions on the basis of a cancer diagnosis such as allowing banks to refuse a mortgage and policy opposition items such as not having a responsibility to provide the best possible care for cancer patients while feeling awkward around someone with cancer was next and only 8-11% agreed with personal responsibility statements, such as blaming a person with cancer condition. This has been concurred by the paper of Justine et al. [23], wherein the severity of a cancer diagnosis came at the top with the highest level of agreement whereas ‘avoiding someone with cancer’ showed the least agreement. Consequently, in a qualitative study done by Nyblade et al. [30], three main components emerged for cancer stigma driving manifestations such as fear of casual transmission of cancer, personal accountability for having caused cancer, and belief in and fear of the certainty of disability and death with a cancer diagnosis. Stigma causes negative effects on patients' mental health which may have been a barrier to screening, early diagnosis, and treatment. Thus, it is important to monitor patients who are showing signs of stigma and provide necessary interventions to reduce their negative thoughts toward cancer condition.

Data in this study indicates that increasing age was associated with an increasing level of cancer stigma. This is partially contradicted by the report of Justine et al. [23], who reported that younger students were estimated to have a higher chance of having increased cancer stigma levels; other significant predictors being reported were male gender, junior students, non-health science students, and those without a family history of cancer. This had also been observed byErnst L [31], wherein age, males, lack of cancer-related experiences, and the assumptions of not being able to protect from cancer were the strongest predictors of cancer stigma. In our study, we also observed male gender as the significant factor of stigma; however, we found no significant association between cancer stigma in terms of education and family/friend history of cancer. In China, a study carried out among breast cancer survivors found that participants with multiple comorbidities had a higher chance of suffering from cancer stigma than those without comorbidities. The author emphasized the need to strengthen the management of stigma among breast cancer survivors, especially among survivors with associated comorbidities. Studies showed that cancer stigma is a leading cause of delayed cancer screening among the general population. Qualitative studies conducted in the US and Australia found cancer stigma as a barrier to lung cancer screening and seeking help for potential lung cancer symptoms [32,33]. Greater overall cancer stigma in the UK was strongly linked to a lower chance of cervical, breast, and colorectal cancer screenings [31]. In a study conducted in India, women with cervical and breast cancer reported that stigma surrounding the disease was present in their lives and communities and this prevented them from performing cervical cancer screening and treatment even at the advanced stages of the disease [27].

We conclude from the above studies that one of the reasons for delayed detection and treatment of cancer is the stigma around the disease. For instance, one study showed that breast cancer detection delay is highly associated with poor prognosis [34]. In Saudi Arabia, breast cancer carries a poor prognosis due to delays in diagnosis and start of treatment. This emphasizes the importance of early cancer detection by appropriate screening [35,36]. In the end, further assessment of the driving factors of cancer stigma is warranted.

Limitation

This study has several limitations to consider. First, the use of social media to distribute the questionnaire could introduce a sampling bias, as it predominantly captures the views of those active on these platforms, potentially excluding segments of the population not engaged with social media. Second, given its cross-sectional design, the study only provides a snapshot at a specific point in time, making it challenging to determine cause-and-effect relationships or observe changes in attitudes over time.

## Conclusions

Cancer stigma among the Saudi population was generally low. Stigma was more likely associated with older male subjects, but it may not have a direct connection with education or a family history of cancer. These findings set a benchmark of stigmatization among the Saudi general population and may serve as a basis to formulate ideas for cancer stigma treatment. However, further research is needed to explore other driving factors. Such efforts may generate more data, which eventually leads to reduced cancer stigma along with enhanced screening and intervention.
